# The Prevalence of Human T-Lymphotropic Virus Infection among Blood Donors in Southeast China, 2004-2013

**DOI:** 10.1371/journal.pntd.0003685

**Published:** 2015-04-01

**Authors:** Jinzhen Xie, Shengxiang Ge, Yali Zhang, Yongcai Lin, Hongying Ni, Jun Zhang, Changrong Chen

**Affiliations:** 1 Xiamen Blood Services, Xiamen, P.R. China; 2 State Key Laboratory of Molecular Vaccinology and Molecular Diagnostics, Xiamen, P.R. China; 3 National Institute of Diagnostics and Vaccine Development in Infectious Diseases, Xiamen, P.R. China; 4 Collaborative Innovation Center of Biological Products, Xiamen, P.R. China; 5 School of Public Health, Xiamen University, Xiamen, P.R. China; University of Florida, UNITED STATES

## Abstract

**Background:**

The human T-lymphotropic virus type 1 (HTLV-1) which is associated with the diseases of adult T-cell leukemia/lymphoma, HTLV-1 associated myelopathy / tropical spastic paraparesis (HAM/TSP) and HTLV-associated uveitis, can cause transfusion-transmitted infections. Although HTLV screening of blood donors was already routinely performed in developed countries, little is know about the HTLV prevalence among blood donors in developing countries which do not perform HTLV screening, such as China.

**Objectives &Aims:**

To systematically characterize the prevalence of HTLV infection among bloods in southeast China.

**Methods:**

A 10-year survey for HTLV prevalence in blood donors was performed in Xiamen, southeast China, during 2004-2013. The HTLV-1/2 of blood donations were screened by enzyme-linked immunosorbent assay, following with confirmation by western blot assay and 9nucleic acid testing. The HTLV-1 prevalences in donors from different cities were calculated. Viral sequences derived from identified HTLV-positive cases were sequenced and analyzed.

**Results:**

Among 253,855 blood donors, 43 were confirmed to be seropositive for HTLV-1 (16.9 per 100,000 95% CI: 12.3-22.8) and none HTLV-2 infection was found. The HTLV-1 prevalence varied significantly in donors from different cities. Donors from cities in Fujian province (24.3 per 100,000, 95%CI: 17.4-33.1) had a significantly higher (p=0.001) HTLV-1 seroprevalence than those who were born in non-Fujian cities (3.4 per 100,000, 95%CI: 0.7-9.8). Among nine cities in Fujian province, the highest prevalence was found in blood donors from Ningde (171.3 per 100,000, 95%CI: 91.3-292.8) which is a coastal city in the northeast of Fujian. Molecular characterization of viral sequences from 27 HTLV-1 carriers revealed 25 were Transcontinental subtype of genotype A and 2 were Japanese subtype of genotype A. Interestingly, 12 of 25 Transcontinental subtype sequences harbored a characteristic L55P mutation in viral gp46 protein, which was only presented in the Transcontinental subtype sequences from Japan and Taiwan but not in that from other countries.

**Conclusions:**

Although China is considered to be a non-endemic region for HTLV, the HTLV-1 prevalence in blood donors is significantly higher in Fujian province, southeast China. A higher prevalence of HTLV-1 in the Fujian may be attributed to endemic foci in the city of Ningde.

## Author Summary

The human T-lymphotropic virus type 1 (HTLV-1) which is associated with the diseases of adult T-cell leukemia/lymphoma, tropical spastic paraparesis etc., can cause transfusion-transmitted infections, it also can be transmitted by sex or breastfeeding. Globally, approximately 20 million people are estimated to be infected by HTLV-1, and 90% of them remain asymptomatic carriers during their lives. Previous studies had revealed that Japan, Central and Western Africa, the Caribbean islands and Central and South American were the regions with the highest HTLV-1 prevalence in the world. Little is know about the HTLV prevalence in China. We performed a 10-year blood screening survey to systematically characterize the prevalence of HTLV infection among bloods in Fujian province in southeast China since 2004. The HTLV-1 prevalence in blood donors is significantly higher in southeast China, especially in the northern cities of Fujian province, such as Ningde. Moreover, similar molecular characteristics of prevalent HTLV-1 sequences in southeast China, Taiwan and Japan suggested a same origin of these viruses.

## Introduction

Human T-lymphotropic virus (HTLV) is a retrovirus which has been known for about 35 years since it was first isolated from a patient with a T-cell malignancy [1]. Previous studies had revealed the etiologic role of HTLV infection in the diseases of adult T-cell leukemia/lymphoma (ATL), HTLV-1 associated myelopathy / tropical spastic paraparesis (HAM/TSP) and HTLV-associated uveitis (HAU) [2, 3]. There are four HTLV related viruses had been identified: HTLV-1, HTLV-2, HTLV-3 and HTLV-4 [4, 5]. However, only HTLV-1 has been convincingly linked to human diseases at present. The most important routes of HTLV transmission were found to be from mother to child and predominantly through breastfeeding and blood contact [3]. The efficiency of the mother-to-child transmission route is estimated to be 20% [6]. Besides vertical transmissions, horizontal transmissions of HTLV are also usual, possibly resulting from unprotected sex, multiple sexual partners, lifetime contact with an HTLV infected partner and transfusion of blood not tested for HTLV [7]. Compared to other transmission route, intravenous exposure to virus-contaminated blood is the most efficient model of HTLV transmission [7].

Globally, approximately 20 million people are estimated to be infected by HTLV-1, and 90% of them remain asymptomatic carriers during their lives [8]. Previous studies had revealed that Japan, Central and Western Africa, the Caribbean islands and Central and South American were the regions with the highest HTLV-1 prevalence in the world [1]. The seroprevalence in these areas were reported to be higher than 5% of the population tested [3]. Because of the absences of effective treatment options and preventive vaccine for HTLV-associated diseases, current prevention approach of new HTLV infections are mainly depended upon the effective control of viral transmission, either blood transfusion or sexually intercourse. Since 1993, HTLV screening of blood donors was already performed in all developed countries and in some developing countries where the virus is endemic. According to previous epidemiological data, most regions of China were considered as a non-endemic area for HTLV infection [9, 10]. However, the Fujian province in southeast China was found to be a high endemic area for HTLV infection [11]. Hence, we performed a 10-year blood screening survey to systematically characterize the prevalence of HTLV infection among bloods in Fujian province in southeast China since 2004.

## Materials and Methods

### Ethics statement

The study was conducted in accordance with the guidelines of the 1975 Declaration of Helsinki and the principles of good clinical practice. All procedures were approved by the Medical Ethical Committee of Xiamen Blood Services. Written informed consent was obtained from all subjects.

### Blood donors

From 15 February 2004 and 31 December 2013, all voluntary blood donors at the Xiamen Blood Service were regularly tested for HTLV-1/2 infection.

### Serological testing

An anti-HTLV-1/2 enzyme-linked immunosorbent assay (Wantai, Beijing, China) was used for initial screening of the infection. The assays were performed on an ELISA STARlet automated system (Hamilton, Bonaduz, Switzerland) according to the manufacturers’ instructions. The assay had been validated to be sensitive and specific as previously described [11]. All positive samples in the initial assay were repeated for twice using the same assay, followed by a confirmation testing using Western blot (HTLV blot 2.4, Genelabs Diagnostics, SciencePark, Singapore). An HTLV infection episode is defined as positive in both ELISA and Western blot testing.

Other blood screening markers, including hepatitis B surface antigen (HBsAg), anti-human immunodeficiency virus (HIV), anti-*Treponemapallidum* (the syphilisspirochete), and anti-hepatitis C virus (anti-HCV) antibodies were detected by the use of the Murex ELISA products (Abbott Murex, Dartford, UK).

### Nucleic acid testing for HTLV-1

After signing an informed consent, a follow-up samples were collected from all blood donors infected by HTLV. Peripheral blood mononuclear cells (PBMCs) were separated from whole blood and stored at -80°C. Proviral DNA of HTLV-1 in PBMCs were extracted by using QIAamp DNA blood kit (Qiagen, Hilden, Germany), and then were subjected to real-time PCR detection as previously described [12]. Real-time PCR positive samples were further amplified for the coding region of the HTLV-1 gp46 gene (nt5222-nt6151, according to HTLV-1 reference sequence of M33896) by nested-PCR [13]. The PCR products were purified and directly sequenced on an ABI Prism 3130X automatic genetic analyzer(Applied Biosystems).

### Polymorphism analysis of viral sequences

All HTLV-1gp46 sequences obtained in this study and 28 reference sequence in the GenBank were selected for phylogenetic analysis by neighbor-joining with Maximum Composite Likelihood corrected distances in the MEGA4 package, using 1000 bootstrap replicates. The sequences obtained in this study and another 251 HTLV-1 reference sequences derived from different geographical areas and different populations containing viral *env* gene, collected from a public HTLV-1 molecular epidemiology database (http://htlv1db.bahia.fiocruz.br) [14], were analysis for amino acid variability. The new nucleotide sequence data reported in this paper have been deposited in the GenBank databases under the accession numbers (Table 1)

**Table 1:** The GenBank accession numbers.

HTLV-I gp46 sequences	GenBank accession numbers
FJ25-NP	KP666062
FJ26-PT	KP666063
FJ22-PT	KP666064
FJ23-XM	KP666065
FJ16-SM	KP666066
FJ17-SM	KP666067
FJ18-ND	KP666068
FJ20-ND	KP666069
NFJ02	KP666070
FJ19-ZZ	KP666071
FJ21-ND	KP666072
FJ01-QZ	KP666073
FJ02-ND	KP666074
FJ03-ND	KP666075
FJ04-ND	KP666076
FJ05-FZ	KP666077
FJ06-ND	KP666078
FJ07-ND	KP666079
NFJ01	KP666080
FJ08-NP	KP666081
FJ09-ND	KP666082
FJ11-SM	KP666083
FJ10-ND	KP666084
FJ12-PT	KP666085
FJ13-LY	KP666086
FJ14-XM	KP666087
FJ15-ND	KP666088

### Statistical analysis

For donors with multiple donations during the period, only the screening results of the first samples were calculated. Statistical analyses were performed by the Mantel-Haenszel χ2 test and Fisher’s exact test for categorical variables. Differences were considered significant at a 2-tailed p<0.05. SPSS software version 17.0 was used for the all statistical analyses.

## Results

### HTLV prevalence in blood donors

**Fig 1:**
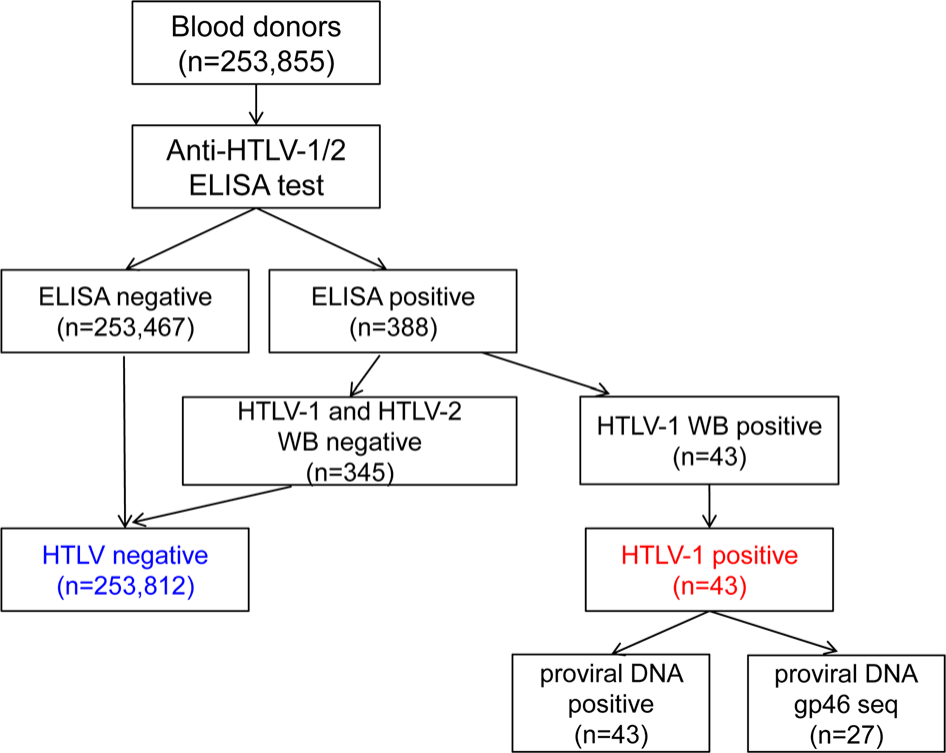
Flow chart of screening and confirmation of HTLV infection.

A total of 253,855 donors were tested for HTLV infection, 43 among them were positive for antibody against HTLV-1, none was positive for anti-HTLV-2, as suggested by ELISA and confirmed by Western blot (Fig 1). All 43 HTLV seropositive donors were also positive in HTLV-1 proviral DNA by real-time PCR. Among them, 2 were co-infected with *Treponemapallidum*, 1 was co-infected with HCV, none was found to be co-infected with HBV or HIV. The overall prevalence of HTLV-1 infection was 16.9 per 100,000 (95% CI, 12.3–22.8).

**Fig 2:**
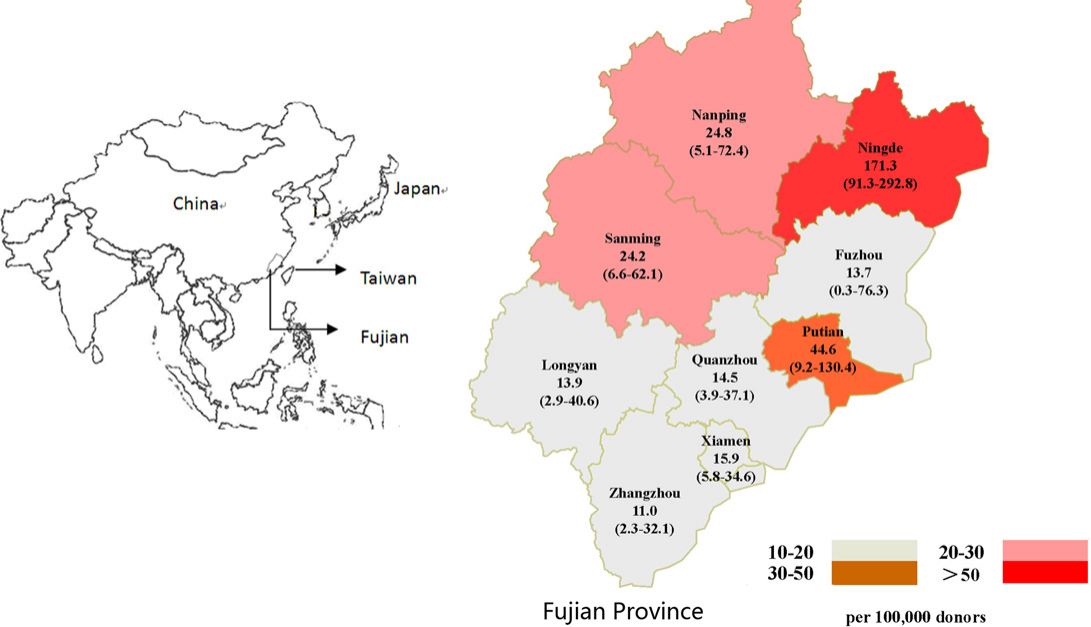
The geographic distribution of HTLV-1 infected donors among Fujian province. The prevalence of HTLV-1 in each city and its 95% CI was showed as 1 / 100,000.

The demographic characteristics of the donors were summarized in Table 2. The HTLV-1 infected donors occurred every year during the study, without significant yearly variance (Table 2). Twenty eight (65%) HTLV-1 carriers were men but the prevalence of men is similar as that of the women. The median age of the carriers is 27.5 (range 18–47, mean 28.4±7.1). The prevalence in donors aged from 26–35 years (26.5 / 100,000) is significantly higher than that of the younger (12.2/100,000). Most of the donors are born in Fujian province (40/43, 93%). The prevalence of Fujianese (24.3/100,000) is 7.24 times (95% CI: 2.24–23.43) higher than that of non-Fujianese (3.4/100,000). The HTLV-1 carries occurred in all nine major cities in Fujian Province, the highest prevalence (171.3 / 100,000) occurred in donors born in Ningde City, a coastal city in the northeast of Fujian (Fig 2).

**Table 2:** Demographic characteristics of the donors.

Characteristics	Donors, No.	Confirmed HTLV (+)			*P* value
		Donors, No.	Prevalence (95%CI) ^a^	Crude OR (95%CI) ^b^	
Overall	253855	43	16.9(12.3–22.8)		
Gender					
Male	144951	28	19.3(12.8–27.9)	1.00	
Female	108904	15	13.8(7.7–22.7)	0.71(0.38–1.34)	0.290
Age, y					
≤25	148148	18	12.2(7.2–19.2)	1.00	
26–35	64135	17	26.5(15.4–42.4)	2.18(1.12–4.23)	0.018
36–45	27094	6	22.1(8.1–48.2)	1.82(0.72–4.59)	0.196
46–55	7168	1	14.0(0.4–77.7)	1.15(0.15–8.60)	0.893
Unknown	7310	1	13.7(0.3–76.2)	1.13(0.15–8.43)	0.908
Year of donation					
2004	23046	7	30.4(12.2–62.6)	1.00	
2005	22979	4	17.4(4.7–44.6)	0.57(0.17–1.96)	0.374
2006	21009	3	14.3(2.9–41.7)	0.47(0.12–1.82)	0.274
2007	23872	3	12.6(2.6–36.7)	0.41(0.11–1.60)	0.201
2008	25325	4	15.8(4.3–40.4)	0.52(0.15–1.78)	0.297
2009	19960	3	15.0(3.1–43.9)	0.49(0.13–1.91)	0.308
2010	30179	7	23.2(9.3–47.8)	0.76(0.27–2.18)	0.614
2011	29432	8	27.2(11.7–53.6)	0.89(0.32–2.47)	0.830
2012	29169	3	10.3(2.1–30.1)	0.34(0.08–1.30)	0.117
2013	28884	1	3.5(0.1–19.3)	0.11(0.01–0.93)	0.033
Place of birth					
Non-Fujian Cities	89452	3	3.4(0.7–9.8)	1.00	
Cities in Fujian	164403	40	24.3(17.4–33.1)	7.26(2.25–23.45)	<0.001
Xiamen	37731	6	15.9(5.8–34.6)	4.74(1.19–18.95)	0.028
Fuzhou	7296	1	13.7(0.3–76.3)	4.09(0.42–39.29)	0.223
Quanzhou	27608	4	14.5(3.9–37.1)	4.32(0.97–19.30)	0.055
Zhangzhou	27279	3	11.0(2.3–32.1)	3.28(0.66–16.24)	0.146
Longyan	21578	3	13.9(2.9–40.6)	4.14(0.84–20.53)	0.082
Ningde	7587	13	171.3(91.3–292.8)	51.15 (14.57–179.6)	<0.001
Putian	6719	3	44.6(9.2–130.4)	13.31(2.69–65.97)	<0.001
Sanming	16496	4	24.2(6.6–62.1)	7.22(1.62–32.30)	0.010
Nanping	12109	3	24.8(5.1–72.4)	7.39(1.49–36.61)	0.051

^a^, Data are percentage of HTLV-positive cases per 100,000 donors;

^b^, Odds ratio, 95% confidence interval

### Molecular characteristics of HTLV-1 in southeast China

**Fig 3:**
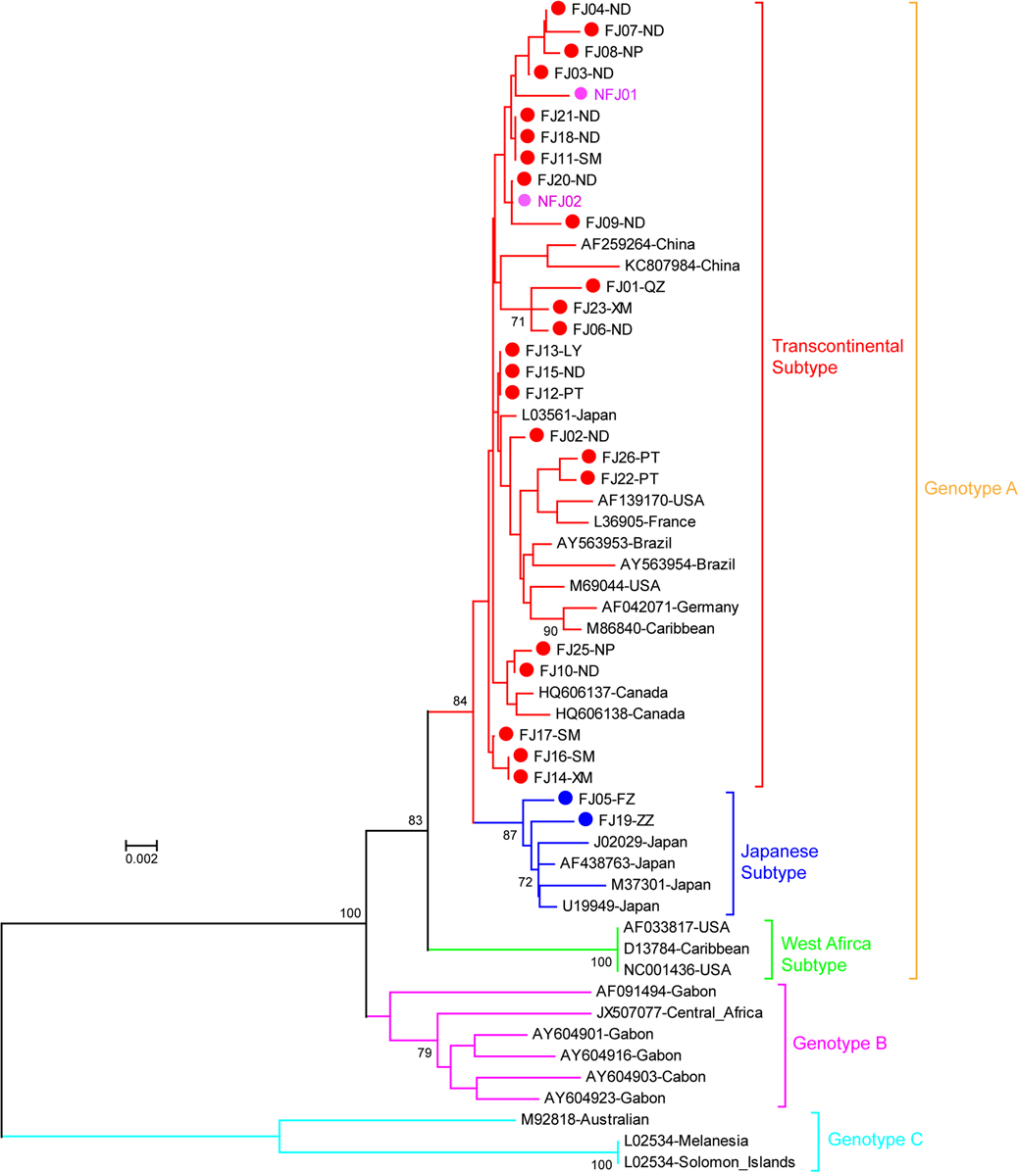
Phylogenetic tree constructed using HTLV-I gp46 sequences by neighbor-joining with Maximum Composite Likelihood corrected distances. Support for the branching order was determined by 1000 bootstrap replicates; only values of 70% or more are shown. For 27 new sequences of this study, 25 sequences of transcontinental subtype were indicated by red (sequences from donors born in Fujian) or purple (sequences from donors born in Non-Fujian province) dots, and 2 sequences of Japanese subtype were indicated by blue dots. FJ, Fujian province; NFJ, Non-Fujian province; FZ, Fuzhou city; XM, Xiamen city; PT, Putian city; QZ, Quanzhou city; ZZ, Zhangzhou city; LY, Longyan; NP, Nanping city; SM, Sanming city; ND, Ningde city.

The HTLV-1 gp46 gene from 27 of the 43 HTLV-1 infected donors were successfully amplified and sequenced. Phylogenetic analysis indicated that 25 sequences (25/27, 92.6%) were clustered into Transcontinental subtype of genotype A and 2 remaining sequences (FJ05-FZ and FJ19-ZZ) were clustered into Japanese subtype of genotype A (Fig 3).

**Fig 4:**
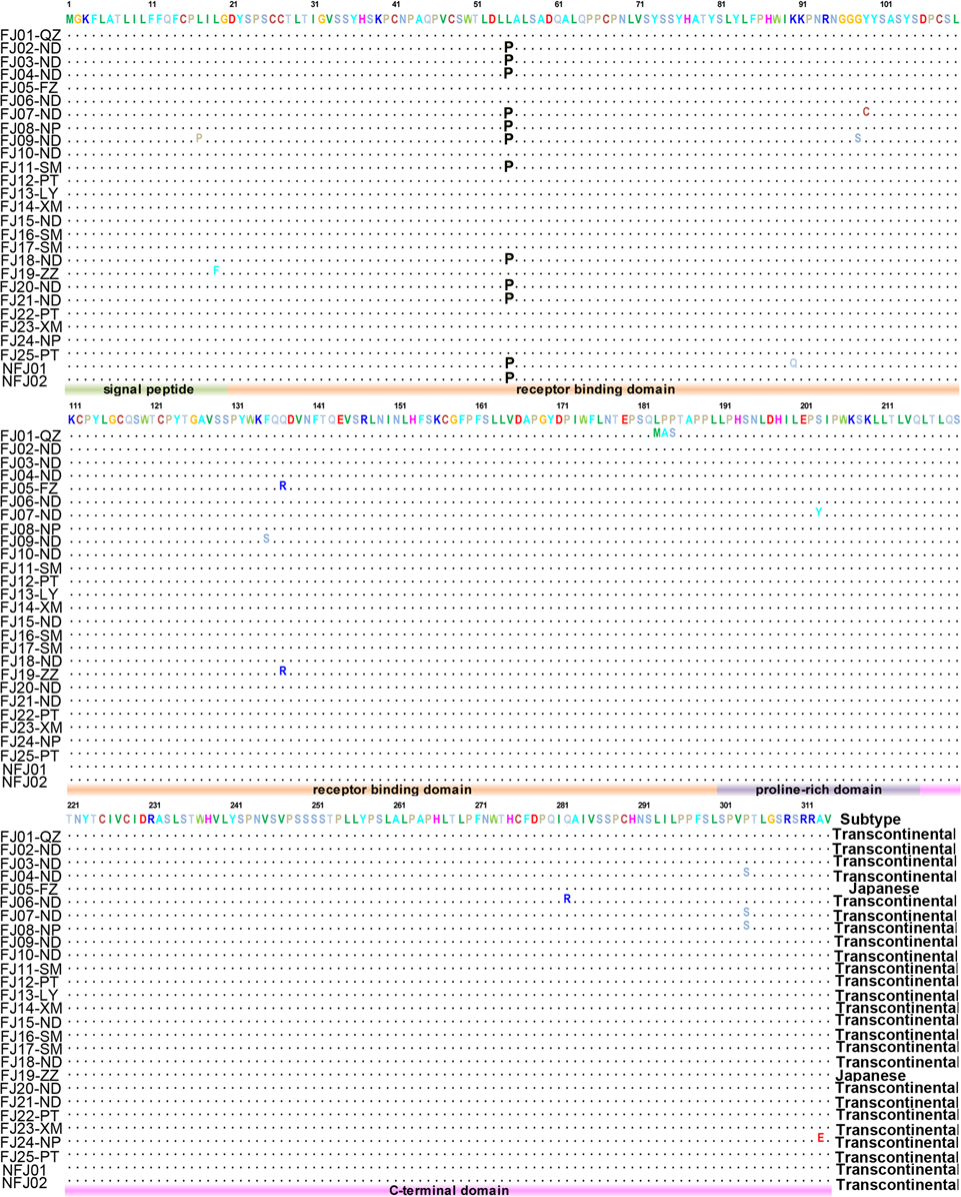
Alignment of amino acids sequences of gp46 gene in strains isolated in the study.

The coded amino acid sequences of the isolated gp46 genes were further analyzed (Fig 4). Ten strains (FJ10-ND, FJ12-PT, FJ13-LY, FJ14-XM, FJ15-ND, FJ16-SM, FJ17-SM, FJ22-PT, FJ23-XM and FJ26-PT) presented identical amino acid sequence with the consensus sequence of HTLV-1 genotype A, whereas the remaining 16 strains harbored at least 1 amino acid substitution. A characteristic L55P mutation in the receptor binding domain (RBD) of gp46 protein [15] was presented in 12 sequences (48.0%) of Transcontinental subtype. To further understand the polymorphism of the L55 site, we collected 251 sequences containing RBD region and their geographic origin information (as shown in S1 Table) from a public HTLV-1 database (http://htlv1db.bahia.fiocruz.br). The comparison indicated that the L55P mutation was only observed in Transcontinental subtype strains originated from Japan (9/20, 45.0%), Taiwan (4/11, 36.4%) and China (1/2, 50%), but not appeared in strains isolated from Transcontinental subtype strains originated from other Asia countries (0/3), Africa (0/6), Central America (0/29), Europe (0/22), North America (0/12), South America (0/38), but also in other subtypes of genotype A (0/67) or genotype B/C (0/40).

## Discussion

This was the first longitudinal and more complete coverage study about HTLV infection of blood donors in China mainland. The key findings include: HLTV-1 infection persist in many cities of Fujian Province, the southeast coast of China. None HTLV-2 was found. The prevalence in blood donors was 16.9 per 100,000 (95% CI: 12.3–22.8), Ningde people up to 171.3(95% CI: 91.3–292.8); molecular analyses demonstrated most of HTLV-1 isolates (25/27, 92.6%) in our study belong to Transcontinental subtype of genotype A; 12 of 25 Transcontinental subtype sequences harbored a characteristic L55P mutation in viral gp46 protein, which was only presented in the Transcontinental subtype sequences from Japan and Taiwan but not in that from other countries.

The HTLV prevalence varies significantly in different geographic areas. The World Health Organization (WHO) has advised that decisions regarding the screening of blood donations for HTLV be guided by local epidemiological evidence. In most Asia countries except Iran and Japan, the HTLV infection appears rare. However, epidemiological data of most areas of Asia, particularly of the China mainland, were very little due to the lack of large-size and representative studies [10]. In China mainland, according the limited reports, the Fujian province in southeast China was suggested to be a relative HTLV endemic region. In 2005, a cross-sectional study reported a total of 19 confirmed HTLV-1 positive cases among 145,293 donors from 13 provinces of China and indicated all 19 HTLV-1 carrier donors were from Fujian province [11]. A recent study conducted in the years of 2012 to 2013 identified 38 confirmed HTLV carriers among 122,468 blood donors from 9 provinces containing 19 blood banks in China. Of the 38 positives, 34 live in Fujian province and the remaining 4 cases were from the Guangdong and Zhejiang provinces that are neighboring to Fujian [10]. We started HTLV screening of blood donors in Xiamen city of Fujian province since 2004 to prevent HTLV associated transfusion-transmitted infection. Through a 10-year survey, as the results presented in this study, we found 43 confirmed HTLV-1 carriers among 253,855 blood donors that indicated a prevalence of 16.9 per 100,000 (95%CI: 12.3–22.8) in our study population. This prevalence was much lower than those in southern Japan, Sub-Saharan Africa, and the Caribbean area, similar with those in Taiwan, Europe and North American [16, 17]. However, one limitation of our study which should be noted that it was conducted in blood donors instead of in general population. The prevalence data derived from blood donors which were subject to selection by the blood center and by self-selection for good health and altruism, introducing bias and likely underestimation of the HTLV prevalence in the general population. Thus, the true HTLV prevalence in general population of southeast China should be higher than that we observed.

Our study revealed that the HTLV-1 prevalence in donors from Fujian province (24.3 per 100,000, 95%CI: 17.4–33.1) was about 7-fold (OR = 7.24, 95%CI, 2.24–23.43) higher than that in donors from non-Fujian provinces (3.4 per 100,000, 95%CI: 0.7–9.8). These data confirmed previous findings regarding Fujian was a HTLV endemic region in China. Furthermore, for the first time, we found the HTLV-1 prevalence significantly varied among donors from nine different cities in Fujian province and it was the highest in donors from Ningde (171.3 per 100,000 95%CI: 91.3–292.8). Statistical analysis demonstrated the HTLV-1 prevalence among donors from Ningde was significantly higher than any other cities of Fujian (p<0.05, respectively), and two neighboring cities (Nanping and Sanming) of Ningde also presented relatively higher prevalence (Fig 2). A new study published recently also noted that donors in Ningde had a very high HTLV-1 prevalence (22/5534), which was consistent with our finding [18]. These data suggested this city was an endemic focus may essentially contribute to the high HTLV-1 prevalence of Fujian region. Further studies should be performed to investigate the risk factors and transmission routes of HTLV-1 infection in these regions, aiming to develop effective control strategies toward HTLV infection.

Molecular analyses demonstrated most of HTLV-1 isolates (25/27, 92.6%) in our study belong to Transcontinental subtype of genotype A. Interestingly, two cases (2/27, 7.4%) carrying Japanese subtype of genotype A were found for the first time in China. Previous study had revealed that the Japanese subtype was a predominant viral strain in some populations of Japan and was also found in neighboring Taiwan [19]. The presence of Japanese subtype in blood donors of southeast China suggested a potential possibility of the introduction of this subtype into southeast China from Japan. In contrast to Japanese subtype, the Transcontinental subtype distributes worldwide. The proportion of the Transcontinental subtype was about 30% in Japan, whereas it was about 70% in Taiwan which is neighboring area of Fujian. Our results demonstrated the HTLV-1 sequences of Transcontinental subtype in Japan, Taiwan and China shared a unique gp46 L55P mutation which was not presented in sequences originated from other geographic regions. This frequencies of the L55P mutation among HTLV-1 in Japan, Taiwan and China were very similar (about 36.4–50.0%), that suggested a close relationship in between Transcontinental subtype viral strains of these regions and these viruses possibly shared a common evolutionary ancestor. Furthermore, potential influence of such a common mutation on viral function phenotype and clinical course certainly warrant further investigation.

In summary, the HTLV-1 prevalence in blood donors is significantly higher in southeast China, especially in the northern cities of Fujian province, such as Ningde. A surveillance system should be implemented to evaluate residual risk of transfusion-transmitted HTLV-1 infection in different regions of China. Moreover, similar molecular characteristics of prevalent HTLV-1 sequences in southeast China, Taiwan and Japan suggested a same origin of these viruses.

## Supporting Information

S1 Checklist STROBE checklist. (DOC)

S1 Table 251 Sequences containing RBD region and their geographic origin information. (XLS)
